# Our New Problem—An Old Idea Enlarged upon

**Published:** 1902-03

**Authors:** F. S. Key Smith

**Affiliations:** Washington, (D. C.) Bar.


### OUR NEW PROBLEM—AN OLD IDEA ENLARGED UPON.

BY F. S. KEY SMITH, LL.M.,

OF THE WASHINGTON, (D. C.) BAR.

The writer had the honor, in the October issue of the *Albany Law Journal*, under the title, "A Study of Rewards as a Remedy for Crime," of presenting to the public a few ideas, which the recent lamented assassination of our president has made him desirous to pursue a shade further with the kind permission of his readers. The righteous indignation of the whole nation, felt and expressed alike by public men, the press and the people, over this most horrible tragedy, is but an evidence of a most healthful sentiment prevalent in the United States, and is, therefore, highly commendable. Now, however, that passion's first impulse has had time to cool, looking dispassionately on the matter, what do we observe? The first to suggest itself is an inquiry: wherefore the cause of anarchy springing up in this land of the free? Surely there must be some reason. Lincoln and Garfield were not shot by anarchists. McKinley is the first. What, then, has sicked these bloodhounds of destruction on the American nation and its people? We must solve the problem, for therein lies the key to the proper solution of how to deal with these monsters. Now that this Grendel has appeared amongst us and taken off one of our greatest citizens, from whence is to come the noble Beowulf, who shall slay both the monster and its mother? Lest the writer should be suspected of attempting to assume that role, he had best assure his readers that nothing is further from his mind, and that his only reason for writing as he does grows out of a sincere conviction of the truth of his views. "Only this and nothing more," as Poe would say.

Since Grendel has shown himself, been captured, convicted and sentenced, the next step of importance is to effectually rid ourselves of his mother. In this, however, without wishing to detract from the courage and bravery of Beowulf, we will not find as easy task as did he. The mother of this Grendel is a monster with many heads and a thousand lives. It can be seen, therefore, it is of the vastest importance that we make no mistake in the methods we employ in dealing with this new evil that has so suddenly sprung up in our midst. A false move at this time, which may require years of diligence in an opposite direction to redeem, would give to anarchy and anarchists such an opportunity for a foothold in the United States that the mistake would be exceedingly hard if not quite impossible to retrieve. The present is no time, therefore, for quibbling, entertaining useless ideas of retaliation, or hanging to worthless, time-worn and inadequate precedents. What is needed is twentieth century ways and highly civilized methods of the broadest type. Striking at the very root of the evil, let us endeavor ascertain the cause of the outbreak of anarchy in the United States. The writer has seen various reasons assigned by the press and public men all over the country. Some allege it to be due to a lawlessness begotten by the lawless spirit which is responsible for lynching. Others argue, with reason, that the depravity in municipal officers and governments have sown the seed and been the inspiration of anarchy in the United States. Certainly the absolutely disgraceful manner in which most of our large cities are governed—the places where anarchists dwell and have their being—is sufficient to convince even those who are not anarchists, if they did not know, that the country at large was not likewise controlled; that governments are not what is claimed for them. To the mind of the writer, however, it would appear that we have been free to the present time from anarchists and their crimes, because, being so unlike foreign powers, they have felt heretofore that they had nothing to fear from the United States; but since our recent development and expansion has placed us in the foremost ranks of the powers of the world, these individuals feel that they are not now quite so sure of their ground in America. Consequently, they begin to look upon us as a new enemy, more powerful than any of their old, which they must immediately destroy, or be themselves, by it, destroyed; hence the assassination of President McKinley, under whose administration these great changes have been wrought. In dealing with these demons, therefore, it is of the utmost importance that we take no steps which could possibly precipitate the prophecy of the Spanish press, that the United States had reached the beginning of her downfall. We must, to prevent this, scrutinize closely, lest we abridge in the least any of the liberties of our people in an attempt to stamp out this horrible crime.

The writer, however, wishes to state that what he has just said has no reference whatever to our policy of expansion, as it has to the present been conducted. But it appears to him as probably the most likely cause for the recent outbreak in this country, and, if he is correct in this, it is manifest that the best and most effectual way to deal with the anarchist is to demonstrate beyond peradvenure, that, while ambitious to possess as a nation all that is good and covetable in the greatest on the globe, we have, on the other hand, neither the desire nor intention of adopting their many vices and oppressions. All this talk, therefore, of a body guard for the president—who is not a ruler, although recently so styled, the people being the sovereign, and their own ruler in this country—as well as the recent utterances of public men and the press relative to making the teaching and preaching of anarchistic doctrine treasonable, when the Constitution has wisely, for well-known reasons, restricted such a crime to levying war against the United States, adhering to its enemies, giving them aid and comfort, is more calculated than any other one thing which we could possibly do to beget anarchy and anarchists in our country. Not longer than five years ago the writer saw it given by the press, when commenting upon either an attempted assassination or an assassination of one of the foreign potentates, as a reason why we did not have anarchists reeking their revenge upon our public men, that permitting them to spout and talk acted as a safety valve which let off the surplus steam, which alone was dangerous.

Whenever a fact is stated, although we may differ on most subjects with him who states it, this should not prejudice us against accepting the truth or allowing it to weigh with us. Mr. Bryan certainly recently hit the nail on the head when he said: "We cannot give full protection to our officials merely by passing laws for the punishment of those who assault them * * * We can only bring absolute security to our public servants by making the government so just and so beneficent that every citizen will be willing to give his life, if need be, to preserve it to posterity * * * Free speech and a free press are essential to free government. No man in public life can object to the publication of the truth, and no man in public life is permanently injured by the publication of a lie. That much is published that should not be is only too evident, but let public opinion correct the evil; that will be more effective than law, and will bring no danger with it. If a paper abuses a political opponent stop your subscription and teach the editor to conduct his paper on respectable lines. There is a sense of justice in the human heart, and he who violates it, violates it at his own peril. This sense of justice ultimately turns abuse to the benefit of the man abused. The present laws against slander and libel are sufficient; leave the rest to a healthy public sentiment—and then help to create the sentiment." (*The Commoner.*) Mr. Albert Shaw, in *The Review of Reviews*, also takes this view of the matter. "After all," says he, "no direct measures taken by national or State law-makers can accomplish very much. The best safeguard lies in our greater devotion as a nation to all the best ideals of a democratic republic. As to the personal safety of our high officers of state, and of other men conspicuous in the world of affairs, we may indeed exercise a little more care; but we cannot provide such safeguards as are thrown about a European monarch without such changes in our methods as are not feasible." And the writer would add, without increasing the tendency towards anarchy.

The writer knows of no law in the United States which makes the belief in anarchy a crime, and he believes there is none. Most certainly he does not think such a law would be either wise, needful or capable of enforcement. What Mr. Bryan, as above quoted, says of slander and libel, is all that is necessary to accomplish everything which may be desired to prevent abuse, and as the punishment for murder is all that could be inflicted for treason, if punishment will deter, our laws are adequate in this direction also. The only remaining inquiry, theretore, to which we have occasion to address ourselves is, does punishment accomplish the ends for which it is inflicted, and should it longer be persevered in? The writer in his previous article has dwelt at length upon this subject, so that it is only necessary here to reaffirm what is there said.

We may kill Czolgosz, but, with his death, will we have killed the mother of his crime? Will she not still live and be the more infuriated by the death of her son? Who, also, can say but what this poor wretch, if permitted to live and properly instructed, might not, especially as he is young and this his first step in crime, develop a fairly good citizen in the end? My reader, do not be too quick to meet this suggestion with the assertion that such a man has neither heart nor appreciation upon which to work, for, in one of our western cities there is a man leading a good and respectable life, who is the brother of Jesse James, and was for years a member of his band of notorious outlaws. This man was never punished, but was placed upon his parole, and, although years have passed, he has never broken that parole. How different it would have been had he served a short term in the penitentiary, will any one deny? Besides, I shall cite as evidence against the correctness of your assertion, my reader, that had Mr. McKinley lived, and Czolgosz been sentenced to ten years' imprisonment in the penitentiary, the chances are ten to one that he would have been released several years sooner for good behavior. This was counted on in speaking of his punishment in that event. I have not had the opportunity as yet to examine into the history of this wise and humane provision of our law, as I some day shall; nevertheless, by its adoption, the large percentage of those released before the expiration of their terms, clearly demonstrates that a criminal is not entirely without appreciation or insensible to reward. It is quite evident, therefore, if properly dealt with, men guilty of crime are capable of being worked with to advantage, and in many cases improved, if proper methods are used. In the great State of New York, within the past ten years, there has been established an institution known as the George Junior Republic, the workings of which are too well known to require elaboration here. A similar institution, modeled after and based upon the New York one, has also, within the past few years, been founded and established in Maryland by the joint efforts of the cities of Baltimore and Washington. Briefly stated, these institutions are intended to replace to a large degree, if not entirely, the reform schools, which have demonstrated their inability to answer the purpose for which they were established, turning away, at the age of twenty-one, more criminals than reformed juvenile offenders. The Junior Republics are what their names would imply, that is, they are small societies within themselves, located out on a farm, away from city temptations and environments, where the pure country air is conducive to healthful morals, minds and bodies. Here, wayward children of both sexes, are sent and taught the various trades and occupations to which they are individually best suited, which are necessary to earn a livelihood; also, the modes and methods of government, having regularly constituted tribunals, executive, legislative and judicial; public officers elected from their ranks by the citizens of the republic at stated intervals. No thought or idea of punishment is for a moment suggested to the children in connection with their confinement in the republic, and consequently their minds are not poisoned against society. They are treated with the utmost civility and kindness, and being made to feel that they are citizens of the republic, are kept pleasantly busy with their duties all the time, which makes the institution, once established, self-sustaining. Thus they live, becoming so infatuated and delighted with their lives and daily work, that their stay goes by with the swiftness and sweetness of a May day, making them reluctant to leave upon reaching the age limit, and doing so invariably with a desire and determination to take the same part in the larger, or real republic, which they have done in the junior. How refreshing is such a system when remembered in comparison with the old-time reformatory.

This paper has failed in its errand, if by now it has not intimated the writer's leaning. He would, therefore, suggest—crude, of course, as are all new ideas before being polished into the perfect stone by the hard rubs of experience, but, it is believed and hoped, beautiful when so polished—that similar treatment be tried with hardened criminals. In a word, when a man has been convicted of crime, send him to an asylum, not for the insane, unless he is insane, but for the treatment of criminals. Such an asylum, presided over and in charge of noted and competent criminologists, who would thus have an opportunity of studying every phase of criminal character and life, could not but go a long way in teaching and directing us in the best methods for the prevention of crimes and the dealing with criminals, if it did not, as the writer thinks it would, improve those so confined. There should be, however, to accomplish this latter end and obtain the best results, no intimation or thought of confinement by way of punishment. Throw all such ideas to the wind and let no one so well understand this as the convict. The object must be to instruct and train the poor unfortunate in his duties towards his fellowman and his country, endeavoring to remove the erroneous idea, prevalent from youth, that society is his bitterest foe, and that every decent man's hand is against him. Lead him, also, to understand that when he has demonstrated an earnest and sincere desire, as well as ability, to conduct himself as he should, and others do, he will be returned to society; employment obtained for him at reasonable wages in any occupation for which he has fitted himself while in the asylum. This will be an incentive for him to labor while there, which, under proper management, can be turned to good account for the State and help to sustain the institution. Such a system will adequately protect the State and country from those who are so steeped in crime as to be incapable of improvement or cure, by providing a safe retreat where they can neither do further harm to society, or marry, to bring descendants of like tendencies into the world to succeed them. Besides this, the released criminal knowing that he is returned to society upon his parole, and that he owes his then employment to the training and influence of the institution which he has left, unlike the criminal under the present system, returned to society with a deeper hatred therefor than before, will not feel that, having compensated society for his crime by serving a term of imprisonment, he is at liberty to commit another just so long as he is willing to abide the consequences if caught. With this the writer is through. He lays down his pen. I wish, however, in conclusion to say, with the twentieth century we have to meet its new conditions and exactions. With our increasing greatness and power which it has thrust upon us, we are obliged to accept and deal with the necessary evils incident thereto, and if anarchy and anarchists be among them, we shall be compelled to deal with these. Let us do so wisely, being careful lest we are unmindful of the experience of foreign nations. Remember, we must, that harsh measures have only served to inflame the passions of these, the worst criminals. In dealing with anarchy we have a subject of too great importance to risk anything which experience has anything like demonstrated to be a mistake. We are obliged now to be sure we are right before going ahead, as our heretofore "any sort of a thing will do" policy will never answer in handling this; both liberty of the press and freedom of speech are at stake, and ere we are aware of the flood-gates being open, as Alexander Hamilton said, "new-fangled and artificial treasons" (*The Federalists*, No. 43), will be the means of our being engulfed in a sea of disorder, tyranny and oppression. If, therefore, our recent calamity is the cause of crime and criminals receiving that attention which they have so long deserved, some good may possibly, after all, come of it, as the Albany *Law Journal*, editorially, in its last issue, points out, saying: "No great crime, such as that at Buffalo, can be committed without bringing some compensative advantages, without teaching lessons, to disregard which would be almost as great a crime as was the original. Naturally, inevitably there has been much ill-considered, foolish talk and not a few ill-digested plans for the nation's succor from this impending danger, both on the part of newspapers and individuals, but out of the many plans for suppressing anarchy before it succeeds in destroying the best and freest government on earth, surely something will come that will be effective."

We can at this time, therefore, only unite in asking of a divine Providence that we be given wisdom, courage and dispassionate judgment in all our deliberations and determinations, so that whatever we do may redound to the glory of Him who hath "made and preserved us a nation," our beloved country and its people.

